# *Corynebacterium striatum* Bacteremia Associated with a Catheter-Related Blood Stream Infection

**DOI:** 10.1155/2017/2682149

**Published:** 2017-01-18

**Authors:** Ueno Daisuke, Tomohiro Oishi, Kunikazu Yamane, Kihei Terada

**Affiliations:** ^1^Department of Digestive Surgery, Kawasaki Medical School, Kurashiki, Japan; ^2^Department of Pediatrics, Kawasaki Medical School, Kurashiki, Japan; ^3^Department of Public Health, Kawasaki Medical School, Kurashiki, Japan

## Abstract

A 49-year-old woman visited our emergency department because of exertional dyspnea due to severe left ventricular functional failure. It progressed to disseminated intravascular coagulation and disturbance of consciousness on day 67 of admission. Gram-positive bacilli were detected from two different blood culture samples on day 67 of admission. An API-Coryne test and sequencing (1~615 bp) of the 16S rRNA gene were performed, and the strain was identified as* Corynebacterium striatum*. The bacterium was detected from the removed central venous catheter tip too, and the patient was diagnosed with catheter-related bloodstream infection by* C. striatum*. However, treatment was not effective, and the patient died on day 73 of admission.

## 1. Introduction

The* Corynebacteria* are a group of aerobic, Gram-positive, catalase-positive, nonsporulating, generally nonmotile rods [1]. The* Corynebacteria* are divided into two groups:* Corynebacterium diphtheriae* and nondiphtherial* Corynebacteria*, collectively referred to as diphtheroids. When isolated from clinical specimens, nondiphtherial* Corynebacteria*, such as* Corynebacterium striatum*,* Corynebacterium amycolatum*,* Corynebacterium minutissimum*,* Corynebacterium xerosis*, and* Corynebacterium freneyi*, were originally thought to be contaminants [2], as these strains are commonly considered as part of the normal flora of human skin and mucous membranes. However, in recent years, they have been reported as emerging opportunistic pathogens in immunocompromised patients with end-stage cancer, hematologic malignancy, and critical condition [2]. There are several reports of* C. striatum* infections including cases of bacteremia, endocarditis, meningitis, pleuropneumonia, osteomyelitis, arthritis, and intrauterine infections [3]. In the present case, we report a catheter-related bloodstream infection caused by* C. striatum*, in a 49-year-old immunocompetent female patient which has multiple organ failures.

## 2. Case Presentation

A 49-year-old woman was brought to our emergency department because of exertional dyspnea due to severe left ventricular functional failure. Her vital signs were unstable; hence, she was immediately admitted to the intensive care unit (ICU). She had two comorbidities: one was diastole cardiomyopathy, and the other was complete atrioventricular block (c-AVB), already treated with a pacemaker implantation (PM).

**Figure 1:**
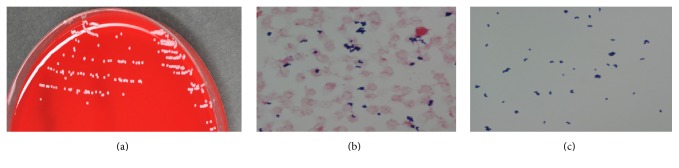
(a) White colonies of 1-2 mm diameter of S-type bacteria, observed after incubation of 5% sheep blood agar at 35°C for 24 hours in carbon dioxide gas culture. (b) Gram-positive coccobacillus revealed by Gram staining in blood sample cultures. (c) Detail of a single colony of Gram-positive bacilli in blood sample cultures.

Although an implantable cardioverter defibrillator (CRT-D, Cardiac Resynchronization Therapy-Defibrillation), with biventricular pacing function, was replaced with PM for severe left ventricular functional decline, on day 12 of admission, an intra-aortic balloon pump (IABP) was also inserted because of multiple organ failure. The IABP was removed on day 16 of admission. Thereafter, there was no obvious fever, signs of infection, so no antibiotics were administered. However, intermittent hemodialysis was continued due to liver failure and renal failure, and an IABP was necessary again after a worsening of cardiac function on day 66. The illness progressed to disseminated intravascular coagulation (DIC) and disturbance of consciousness on day 67 of admission. Therefore, two sets of blood samples for blood culture were collected. Gram-positive bacilli were detected in both blood culture samples; each set included aerobic and anaerobic cultures (Figures 1(b) and 1(c)). A central venous catheter inserted in the patient's right internal jugular vein was removed and the catheter tip was sent for a semiquantitative culture analysis on day 68 of admission. No bacteria species could be identified at this time.

Some asynergy in wall motion was detected by echocardiography, but no vegetation was seen. Initially, on day 67 of admission, tazobactam/piperacillin (TAZ/PIPC) (2.25 g every 6 hours) was prescribed. After the results from blood cultures on day 69 of admission, vancomycin (VCM) (1 g every 6 hours) was added to the therapy, while TAZ/PIPC was changed with meropenem (MEPM) (1 g every 6 hours) on day 72. At this time, another two sets of blood samples were collected and blood cultures were negative. However, the patient died on day 73 of admission.

In order to identify the specific strain of infection, the API-Coryne test (BioMèrieux, France) was performed. This method is based on the assessment of biochemical properties.* C. striatum*/*C. amycolatum* strain was identified with a probability of 89.7%. The nucleotide sequence (1~615 bp) of the 16s rRNA gene revealed a 99.7% homology to a specific subtype, that is,* C. striatum* ATCC 6940 (GenBank: NZ_GG667536). The bacterium was detected from the removed central venous catheter tip too. Thus, the patient was diagnosed with a* C. striatum* catheter-related bloodstream infection. The* C. striatum* strain was susceptible to VCM, linezolid (LZD), and gentamicin (GM) (Table 1).

## 3. Discussion

*C. striatum* colonizes the skin and mucous membranes of both healthy people and hospitalized patients [4]. The majority of cases of* C. striatum* infection are hospital-acquired as wound infections and a few reports on systemic infections [5], that is, infection confirmed by isolation of* C. striatum* from a sterile site, are available. However, most of these cases are represented by patients with implanted indwelling devices or who present an immunosuppression [2, 6]. Because her general condition worsened, implanted indwelling devices as central venous catheter might cause bacteremia by* C. striatum* regardless of patient's history.

To our knowledge, this is the second report which found both blood cultures and cultures from a central venous catheter tip positive for the same strain of* C. striatum* [2]. Since* C. striatum* may have been isolated from blood sample cultures, it is difficult to distinguish an innocuous contamination from a dangerous infection. Outbreaks caused by multidrug-resistant* C. striatum* have been reported in patients with prolonged hospitalization, mechanical ventilation, or use of broad-spectrum antibiotics [7, 8].

Approximately 0.2 to 0.4% of native valve endocarditis is caused by* Corynebacterium* spp., while 9% of early and 4% of late prosthetic valve endocarditis are caused by members of the genus [9, 10]. Although patients with implanted CRT-D may develop infectious endocarditis, obvious vegetation was never observed on echocardiography in these cases [11]. In addition, there is a report of a patient who underwent hemodialysis and developed sepsis caused by a* Corynebacterium* sp. [2]. As the same type of bacteria was detected by the catheter tip culture, the cervical catheter was withdrawn during the hemodialysis, as it was presumed to be the port of entry in this case.

The API-Coryne test is a method to distinguish* C. striatum* from* C. amycolatum*. Although the biochemical properties of* C. amycolatum* and* C. striatum* are similar, only* C. striatum* contains mycolic acid [12]. However, this compound can only be detected by special analyses, for example, gas chromatography; thus* C. striatum* identification was confirmed by 16s rRNA gene analysis.

Since most of reports classified* C. striatum* as susceptible to a wide range of antibiotics [13], it has been suggested that a selective pressure exerted by previous antimicrobial treatment could contribute to its overgrowth. This would eventually lead this strain to become a secondary colonizer in immunocompromised hosts [8].

In general,* C. striatum* is resistant to penicillin but sensitive to other *β*-lactam antibiotics and to vancomycin. In a previous report, vancomycin was recommended as empirical therapy for serious infections caused by* Corynebacterium* spp. [14]. Although* C. striatum* was susceptible to VCM in this case, the patient might have died because administration of VCM was delayed. Therefore, in this case, appropriate antibiotics could not be judged in vivo. However, the optimal antimicrobial therapy for these infections is still controversial. In vitro susceptibility tests showed that linezolid and tigecycline are active against coryneform bacteria, revealing a potential therapeutic value [15, 16] of these compounds. Currently, there are no guidelines for the treatment of* Corynebacterium* spp. infections. Appropriate susceptibility tests and interpretive criteria are critically needed, in light of the growing emergence of multidrug resistance and its involvement in nosocomial infections.

In conclusion, although* Corynebacterium* could be isolated from a blood culture as a common contaminant, in certain case this observation could conceal a dangerous infection. Patients with a history of exposure to broad-spectrum antibiotics or immunosuppression, as well as critically ill patients with an implanted indwelling device or a central venous catheter, must be considered at high risk of severe infection for this type of bacteria and it is necessary to recognize* C. striatum* as an emerging nosocomial pathogen. In conclusion, we encountered a case of catheter-related bloodstream infection caused by* C. striatum*. Unfortunately, we could not successfully treat the patient because of her poor general condition and comorbidity.

## Figures and Tables

**Table 1:** Minimum Inhibitory Concentration of the *C. striatum* strain.

Drug	MIC (*μ*g/mL)
PCG	>2
CTX	>32
CTRX	>2
CFPM	>2
IPM	>8
MEPM	>8
GM	≦0.25
EM	>4
CLDM	>2
MINO	8
VCM	0.5
LZD	≦0.25
CPFX (LVFX)	>4
ST	>38/2

MIC: Minimum Inhibitory Concentration; PCG: benzylpenicillin; CTX: cefotaxime; CTRX: ceftriaxone; CFPM: cefepime; IPM: imipenem; MEPM: meropenem; GM: gentamicin; EM: erythromycin; CLDM: clindamycin; MINO: minocycline; VCM: vancomycin; LZD: linezolid; CPFX: ciprofloxacin; LVFX: levofloxacin; ST: sulfamethoxazole/trimethoprim.
